# Exploration of the omics evidence landscape: adding qualitative labels to predicted protein-protein interactions

**DOI:** 10.1186/gb-2007-8-9-r197

**Published:** 2007-09-19

**Authors:** Vera van Noort, Berend Snel, Martijn A Huynen

**Affiliations:** 1 Centre for Molecular and Biomolecular Informatics, Nijmegen Centre for Molecular Life Sciences, Radboud University Nijmegen Medical Centre, Toernooiveld, 6525 ED Nijmegen, The Netherlands; 2 European Molecular Biology Laboratory, Meyerhofstraße 1, 69117 Heidelberg, Germany; 3 Bioinformatics Group, Department of Biology, Science Faculty, Utrecht University, Padualaan, 3584 CH Utrecht, The Netherlands; 4 Academic Biomedical Centre, Utrecht University, Yalelaan, 3584 CL Utrecht, The Netherlands

## Abstract

**Background:**

In the post-genomic era various functional genomics, proteomics and computational techniques have been developed to elucidate the protein interaction network. While some of these techniques are specific for a certain type of interaction, most predict a mixture of interactions. Qualitative labels are essential for the molecular biologist to experimentally verify predicted interactions.

**Results:**

Of the individual protein-protein interaction prediction methods, some can predict physical interactions without producing other types of interactions. None of the methods can specifically predict metabolic interactions. We have constructed an 'omics evidence landscape' that combines all sources of evidence for protein interactions from various types of omics data for *Saccharomyces cerevisiae*. We explore this evidence landscape to identify areas with either only metabolic or only physical interactions, allowing us to specifically predict the nature of new interactions in these areas. We combine the datasets in ways that examine the whole evidence landscape and not only the highest scoring protein pairs in both datasets and find specific predictions.

**Conclusion:**

The combination of evidence types in the form of the evidence landscape allows for qualitative labels to be inferred and placed on the predicted protein interaction network of *S. cerevisiae*. These qualitative labels will help in the biological interpretation of gene networks and will direct experimental verification of the predicted interactions.

## Abstract 2

By combining different types of large datasets that give evidence for protein-interactions, qualitative labels on the predicted protein interaction network of S. cerevisiae could be inferred, providing guidance towards direct experimental verification of the predicted interactions.

## Background

Genome sequencing projects have resulted in the listing of all protein coding and RNA genes for a large number of organisms. In order to understand how the inner workings of the cell, a plethora of omics (genome-scale) techniques that measure the functional coupling between all the components has been developed. All these techniques measure different aspects of functional coupling: for example, yeast-two-hybrid assays [1,2] uncover direct physical interactions between proteins, whereas affinity purification [3,4] measures the tendency for proteins to be members of the same protein complex, and micro-arrays [5] detect the concerted expression of genes at the mRNA level. Furthermore, functional relationships are predicted from many other sources: genetic interaction data [6], gene fusion, conserved gene neighborhood and gene co-occurrence [7-9], conserved co-expression between species [10,11] or the sharing of transcription factors [12]. Many of these high-throughput techniques to infer functional relationships produce noisy data. The noise level of the data has lead to the development of bioinformatics data integration strategies to increase the reliability of the prediction of functional coupling.

Despite the obvious success of these integrative approaches, they remove from the raw data the information pertaining to functional coupling that was measured in the original assay; high quality generic gene networks have been inferred from the integration of very heterogeneous data, such as synthetic lethals, yeast-two-hybrid and mRNA derived co-expression [13,14]. These networks contain many accurate predictions, but specific information on the type of functional coupling is lost. In addition to the loss of specificity from integration, some techniques to measure interactions, such as co-expression, predict, even without integration, only generic functional couplings. This lack of specificity is a problem, because for the biological interpretation of gene networks and the prioritization of experimental verification, we not only need to identify protein interactions, but also to add qualitative labels to the interactions [15]. We here present a bioinformatics approach that distinguishes different types of functional coupling on the basis of their behavior across different high-throughput datasets. We study how well *in silico *predictions and omics data serve to specifically predict a specific type of interaction. Subsequently, we combine the information from *in silico *predictions, functional genomics data and protein interaction assays into evidence landscapes. In these landscapes we identify regions that are populated solely by physical or metabolic interactions, allowing specific prediction of the nature of interactions between proteins.

### Reference sets

**Figure 1:**
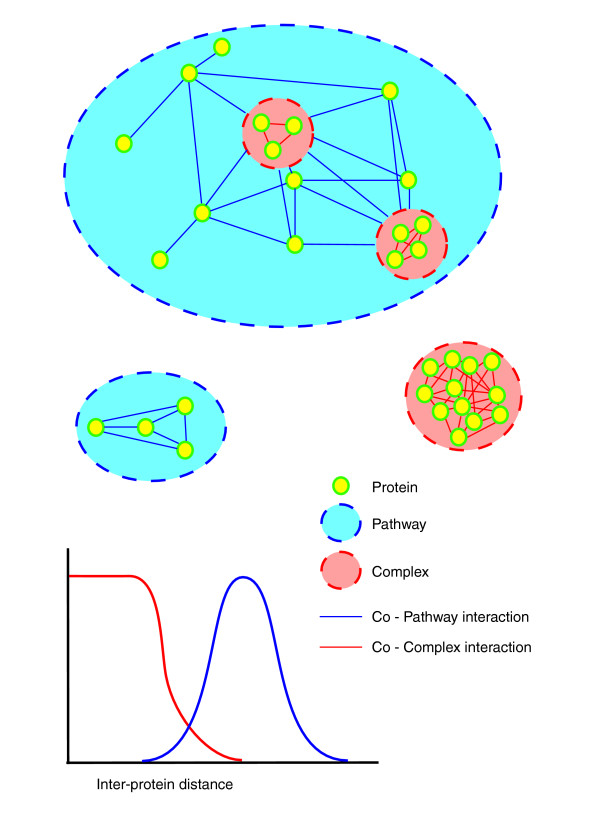
Two categories of functional relationships. The two categories of functional relationships that we use in this study are physical interactions (specifically co-complex memberships) and metabolic interactions. The physical interactions (red) exist between proteins that are identified in the same protein complex, whereas the metabolic interactions (blue) exist between proteins that act in the same metabolic pathway. Metabolic interactions may also exist between individual members of a complex and proteins that act in the same pathway as this complex. One aspect of the nature of these to functional relationships is the physical distance between proteins as illustrated in the graph. As the nature of the relationships differs, one might expect differential behavior in high-throughput experiments.

We chose to analyze omics datasets for the budding yeast *Saccharomyces cerevisiae *because of the availability of much high quality genomics data as well as classical knowledge about its protein functions. We compiled reference sets that are specific for a category of interactions. Although a myriad of functional couplings exist, we here begin by defining two categories that are themselves widely used in data integration studies (Figure 1). Our first and most straightforward category is a physical interaction that is mediated by physical proximity (red lines connecting proteins in Figure 1). A subclass of this category is co-complex membership, which is well defined and for which trustworthy and large reference sets are available, in contrast to direct pairwise physical interactions. We thus used known complexes from the MIPS database as a basis for physical interactions. Care was taken to remove potential circularity in the form of those entries in the MIPS database that are explicitly based on the large-scale protein complex purification data. This resulted in 14,988 positives and 884,224 negatives for pairwise interactions.

Co-pathway membership is another common functional relationship that is frequently used [13,14] and which we chose as our second category. Metabolic interactions, in which proteins are part of the same metabolic pathway, are the clearest exponent of these pathway interactions for which clear cut databases of sufficient size are available. No high-throughput method exists that exclusively detects pathway or metabolic interactions, even though certain methods detect them among other functional relationships. As a basis for metabolic interactions we took only those KEGG maps that represent metabolic pathways (that is, with map number below 2000); obviously, metabolic pathways contain multimeric enzyme complexes, but we did not score the intra-complex interaction of these as positives or as negatives in our metabolic reference. We did, however, consider the links between these enzymes and other enzymes from the pathway as metabolic (Figure 1). This resulted in 18,460 positives and 275,768 negatives.

### Score-intervals and positive predictive value

**Figure 2:**
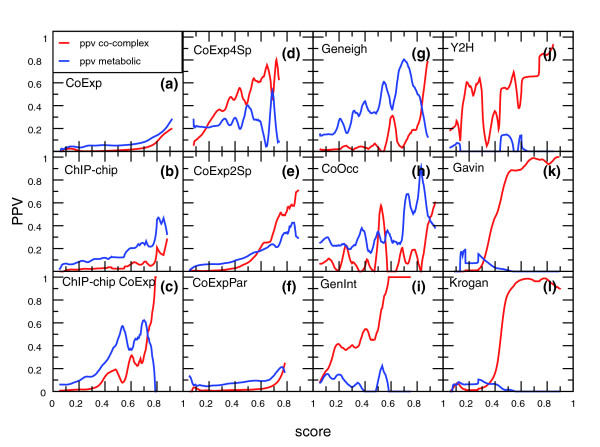
Score-ppv plots of individual datasets. On the x-axis is the score for that dataset, on the y-axis the ppv. The ppv was calculated in all score intervals with bin-width 0.025. Red lines indicate ppv on the protein complex reference set, being the number of true positives in the complex reference set divided by the number of true positives and false positives in both reference sets. Blue lines indicate the ppv on the metabolic reference set, being the number of true positives in the metabolic reference set divided by the number of true positives and false positives in both reference sets. **(a) **Correlated mRNA expression (CoExp). **(b) **Shared binding of transcription factors (ChIP-chip). **(c) **Co-regulation (ChIP-chip*CoExp). **(d) **Conserved co-expression between four species (CoExp4Sp). **(e) **Conserved co-expression between two species (CoExp2Sp). **(f) **Paralogous conserved co-expression (CoExpPar). **(g) **Gene neighborhood conservation (GenNeigh). **(h) **Correlated phylogenetic profiles (CoOcc). **(i) **Shared genetic interactions (GenInt). **(j) **Yeast-two-hybrid (Y2H). **(k) **TAP-tag purifications (Gavin *et al*. [3]). **(l) **TAP-tag purifications (Krogan *et al*. [4]). For (k, l) the protein pairs that are never co-purified and thus have a SA score of 0 are in bin 0.2.

We chose to measure the performance based on score intervals as opposed to thresholds to measure prediction performance. Widely used measures, such as false negative rate or receiver operating characteristic (ROC) curves divide predictions into positives (higher than a threshold) and negatives (lower than a threshold) and then score true and false predictions. Thus, they rely heavily on the assumption that interactions that score higher than a certain threshold are the true interactions. A performance measure based on score intervals does not rely on such assumptions. In contrast, it can identify score intervals with high predictive performance anywhere along a scoring axis and is better suited for integration of data types with different noise levels [14]. A performance measure that combines well with a score interval based scheme is the positive predictive value (ppv). As the predictions are not divided into positive and negative, there are no true and false negative rates. Just like the likelihood ratio in Bayesian networks [14], the ppv depends only on positive predictions without implying that all the other predictions are negative. Figure 2 reflects that indeed some of the evidence types show a high ppv for metabolic interactions at intermediate scores whereas this ppv drops at higher score intervals, which would not have been observed by a threshold based performance measure.

To determine whether there are omics evidence type data that alone are typical for either of the two categories, we cannot simply plot the prediction performance for each reference set independently. We have to take into account true and false metabolic interactions as false physical interactions and vice versa. The ppv of metabolic interactions is calculated as the total number of true metabolic interactions divided by the sum of the true and false metabolic and the true and false physical interactions. The ppv of physical interactions is then calculated as the total number of true physical interactions divided by the sum of the true and false metabolic and the true and false physical interactions. By doing this, we can determine not only whether at a certain score in a certain dataset proteins are likely to interact, but also how they interact.

## Results

### Qualitative information from individual omics datasets

We calculated the ppv for each omics evidence type and each score interval. Figure 2 shows at what score each evidence type successfully predicts either metabolic or physical interactions. The ppv for physical interaction (ppv *phys*) increases similarly to the ppv for metabolic interactions (ppv *meta*) for gene co-expression (CoExp), as well as for combinations of gene co-expression between species (CoExp2Sp, CoExp4Sp) and the combination of gene co-expression with shared transcription factor binding sites (ChIP-chipCoExp) (Figure 2). These data are, therefore, not specific for either metabolic or physical interactions. In contrast, for gene neighborhood (GenNeigh) the ppv depends on the score: very high is specific for physical interactions whereas a lower, but still significant, score is indicative of a metabolic interaction (Figure 2, GenNeigh). The highest ppv *meta *in this set is 0.79, at a point where the ppv *phys *is 0.05, whereas the highest ppv *phys *is 0.73 when the ppv *meta *is 0.11. Therefore, GenNeigh can be used to obtain some specificity about the type of predicted interaction. Correlated phylogenetic profiles (CoOccur) show a similar, but less pronounced, trend of differential ppv.

To get statistical support for these visually observed trends, we employed logistic regression where the binary dependent variable is the presence/absence of an interaction and the continuous variables are the scores from the omics data (Table 1). For all measures of co-expression, the logistic regression co-efficients are positive and significant for both physical and metabolic interactions. The logistic regression coefficients for GenNeigh and CoOccur for the metabolic interactions are not very high due to the probabilities of metabolic interactions not following a logistic curve. Finally, we observe specificity for physical interactions not only in datasets where physical interaction was measured directly (yeast-two-hybrid and protein complex purifications), but surprisingly, also in one that contains a number of shared genetic interactions between proteins (GenInt). With regard to the socio-affinity (SA) score based on the protein complex purifications of Gavin *et al*. [3] and Krogan *et al*. [4], it was expected that a high score in either of these sets would be indicative of a physical interaction. Similarly, the logistic regression coefficient is very high, while for these datasets the regression coefficient for metabolic interactions is not significant. In conclusion, we can specifically pinpoint physical interactions based on single 'omics' datasets, both from visual inspection of the score-ppv plots as well as from the results of the logistic regression.

**Table 1:** Logistic regression coefficients with metabolic and physical interactions

Input	Intercepts	Coefficients	R^2 ^value
**CoExp**			
Metabolic	-5.01*	2.44*	0.00766
Physical	-8.82*	7.25*	0.0588
**ChIP-chip**			
Metabolic	-3.37*	0.568^†^	0.000603
Physical	-4.95*	2.11*	0.00666
ChIP-chip*CoExp			
Metabolic	-4.32*	5.02*	0.0246
Physical	-6.39*	9.17*	0.0745
**CoExp4Sp**			
Metabolic	-2.32*	2.36^†^	0.00706
Physical	-2.97*	6.33*	0.0546
**CoExp2Sp**			
Metabolic	-4.02*	2.55*	0.00594
Physical	-8.16*	10.5*	0.103
**CoExpPar**			
Metabolic	-2.62*	-2.07*	0.00484
Physical	-7.48*	6.17*	0.0373
**GenNeigh**			
Metabolic	-2.69*	2.96*	0.0280
Physical	-5.65*	6.20*	0.219
**CoOcc**			
Metabolic	-1.71*	1.49*	0.0223
Physical	-3.69*	3.27*	0.120
**GenInt**			
Metabolic	-4.18*	4.06*	0.0120
Physical	-3.16*	11.3*	0.113
**Y2H**			
Metabolic	-3.35*	-3.89*	0.106
Physical	-2.30*	4.29*	0.119
**TAP-tag Gavin**			
Metabolic	-3.85*	-0.153	1.87e-06
Physical	-10.3*	24.5*	0.298
**TAP-tag Krogan**			
Metabolic	-3.68*	0.0350	9.19e-08
Physical	-8.99*	18.37*	0.146
**TAP-tag (G+K)**			
Metabolic	-3.78*	-0.54	1.77e-05
Physical	-12.3*	32.7*	0.322

The scores of the 'omics' datasets were in turn considered as the continuous independent variable to fit a logit function to the presence/absence of interactions. *P < 2e-16; ^†^*P *< 0.0001. CoExp, correlated mRNA expression; ChIP-chip, shared binding of transcription factors; ChIP-chip*CoExp, co-regulation; CoExp4Sp, conserved co-expression between four species; CoExp2Sp, conserved co-expression between two species; CoExpPar, paralogous conserved co-expression; GenNeigh, CoOcc, correlated phylogenetic profiles; GenInt, shared genetic interactions; Y2H, yeast-two-hybrid; TAP-tag Gavin, TAP-tag purifications (Gavin *et al*. [3]); TAP-tag Krogan, TAP-tag purifications (Krogan *et al*. [4]); TAP-tag (G+K), the sum of SA scores derived from the two TAP-tag purification data sets.

### Qualitative information from evidence landscapes

Normally, logistic regression provides the best fitting function between a dependent variable and a set of independent variables. In this case the variables are clearly not independent, as can be readily observed in a correlation matrix (Additional data file 1). There are also huge differences in coefficients between fitted functions on the separate variables and fitted functions on multiple variables at the same time (data not shown). Therefore, a simple logistic regression, for example, as applied in [16], is not permitted by these data. Moreover, the interval score-ppv plots show that the probabilities of interactions do not always follow a logistic curve. An exploration of the combinations of scores of the different input data is more suitable. We call the combinations of omics data 'evidence landscapes', surfaces on which the x and y coordinates represent the scores of two types of 'omics' data. In these areas we plot the specificity for either metabolic or physical interactions, estimated by the differential ppv. The differential ppv is computed by subtracting the physical interaction ppv from the metabolic interaction ppv. This means that if a region scores equally well in both reference sets (be it very poor or very well), it has a zero differential ppv, reflecting the inability of this region to differentiate between metabolic and physical interactions. However, if it is very accurate in predicting metabolic relations but unable to accurately predict physical interactions, it has a very high differential ppv and, vice versa, a very negative value reflects specificity for physical interactions. Thus, the differential ppv is a tool to judge whether areas exist that specifically predict either type of interaction.

**Figure 3:**
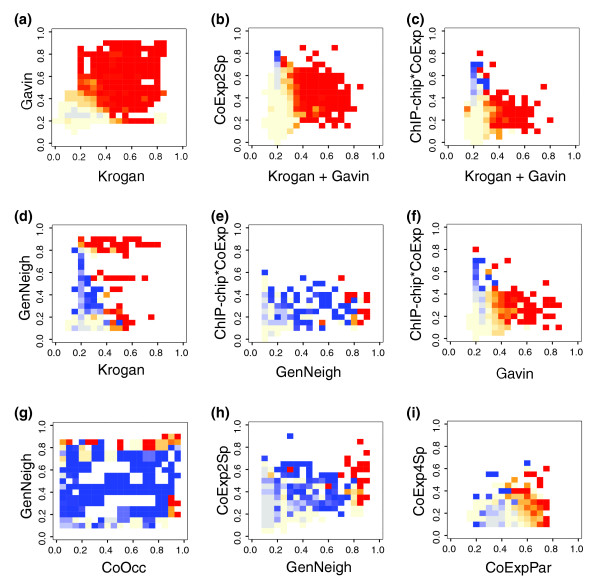
Differential ppv in the evidence landscape. In each panel the x-axis indicates the score in the first dataset, the y-axis the score in the second set. The color scheme is based on differential ppv, being the ppv on the metabolic reference set minus the ppv on the physical interaction reference set. Differential ppv 1 is dark blue, 0 is yellow and -1 is red, parts that contain no gene pairs are white. The blue parts of the landscapes are regions where there are only metabolic interactions, whereas in the red parts there are only physical interactions. **(a) **TAP-tag purifications (Krogan) versus TAP-tag purifications (Gavin). **(b) **TAP-tag purifications (sum Krogan Gavin) versus conserved co-expression (CoExp2Sp). **(c) **TAP-tag purifications (sum Krogan Gavin) versus co-regulation (ChIP-chip*CoExp). **(d) **TAP-tag purifications (Krogan) versus gene neighborhood conservation (GenNeigh). **(e) **Gene neighborhood conservation (GenNeigh) versus co-regulation (ChIP-chip*CoExp). **(f) **TAP-tag purifications (Gavin) versus co-regulation (ChIP-chip*CoExp). **(g) **Correlated phylogenetic profiles (CoOcc) versus gene neighborhood conservation (GenNeigh).**(h) **Gene neighborhood conservation (GenNeigh) versus conserved co-expression (CoExp2Sp). **(i) **Paralogous conserved co-expression (CoExpPar) versus conserved co-expression (CoExp4Sp).

Figure 3 shows the differential ppv in a representative selection of these evidence landscapes. The comprehensive collection of all evidence landscapes is available at our webpage [17]. Figure 3a shows the evidence landscape of the two TAP-tag protein-protein interaction datasets [3,4]. Despite the very high quality of both datasets, they are not completely comprehensive; each dataset identifies interactions with a high SA score [3] between proteins that in the other assay were never co-purified, but which are true interactions in the physical interaction reference set. An SA score of 5 (bin 0.4) in only one of the two assays is not enough to predict a reliable physical interaction; however, if the protein pair has an SA score of 5 in both sets, it is a reliable prediction. So in fact the two assays complement each other. That is why we used the sum of the two SA scores for the evidence landscapes with other 'omics' sets (Figure 3b, c); in these panels, bin 0.2 contains all protein pairs that were purified in both assays but never co-purified. Gene pairs with high orthologous conserved co-expression (CoExp2Sp) that were never co-purified are purely metabolic interactions (the upper left corner of Figure 3b). We also observe this for co-regulated gene pairs (ChIP-chip, Figure 3c). Indeed, we are now able to predict purely metabolic interactions by taking gene pairs that have a null score in the physical interaction set and a positive score for co-expression or co-regulation. Gene-pairs that are null scoring in the complex purification datasets and have an intermediate score in gene neighborhood or correlated phylogenetic profiles also define purely metabolic interactions.

What we have observed in Figure 2 is that intermediate scores in correlated phylogenetic profiles and gene neighborhood conservation are often indicative of metabolic interactions. The evidence landscape of these two has specific metabolic interactions in intermediate scores of both sets (Figure 3g). Thus, not only do we find purely metabolic interactions from gene pairs that score null in protein-protein interaction datasets, we also find them in overlaps with intermediate scoring parts of other evidence types.

### A cellular network with qualitative labels on the predicted interactions

**Figure 4:**
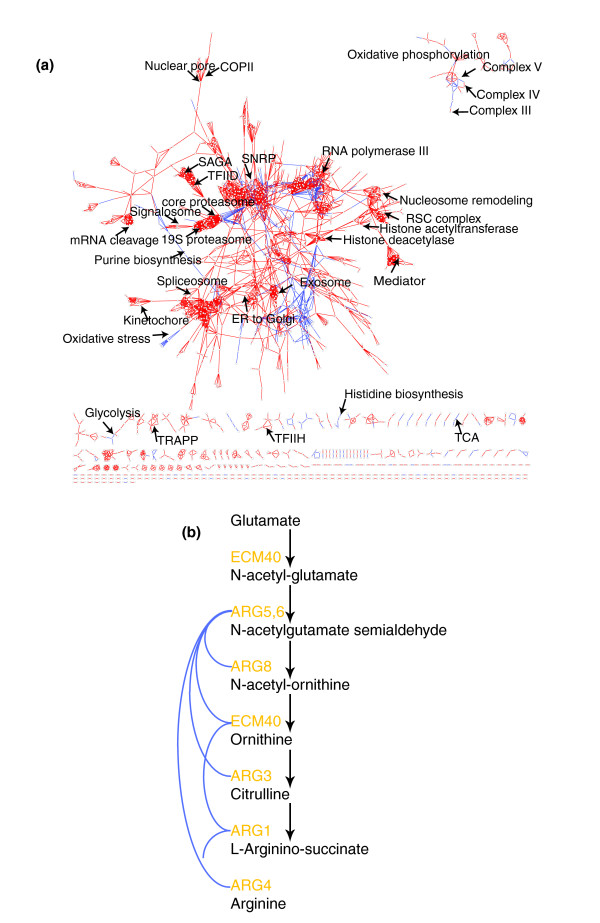
Network with qualitative labels on predicted interactions. **(a) **The network of interactions in yeast that are specifically predicted to be physical (red lines) or metabolic (blue lines). We took all gene pairs that fell into squares (Figure 3) with a differential ppv larger than 0.95 and at least five true positive metabolic interactions for the specific metabolic interactions. We selected all gene pairs that fell into squares with differential ppv smaller than -0.95 and at least five true positive physical interactions for the specific physical interactions. Names of several known complexes and metabolic pathways are indicated on the network. **(b) **The arginine biosynthesis pathway in yeast. Names of the enzymes are in orange, arrows indicate biochemical reactions. Blue lines indicate all interactions that exist for these genes. Note that ECM40 catalyzes two steps in this pathway but the interactions with the other genes are drawn only once.

We extracted a list of predicted metabolic and physical interactions by taking all gene pairs from areas in all evidence landscapes where the differential ppv is either higher than 0.95 or lower than 0.95. We predicted novel metabolic and physical interactions by taking protein pairs that do not overlap with the reference sets but have the same combinations of scores in the evidence landscape. In total, we retrieved 812 of the metabolic interactions in the reference set and 6,996 of the physical interactions in the reference set. Additionally, we predicted 2,985 new physical and 140 new metabolic interactions. This allows us to display a network of physical (red) and metabolic (blue) interactions (Figure 4a). All predicted interactions are available at our webpage [17]. Network visualizations are generally more open to biological interpretation than long lists of potential interactions. It is directly clear from the network layout that physical interactions are more clustered than metabolic interactions. The clustering coefficient (fraction of indirectly connected proteins that are also directly connected) of physical interactions (0.53) is much higher than the clustering coefficient of metabolic interactions (0.031). The incompleteness of the metabolic network relative to the physical interaction network may bias this difference. However, the average number of connections per protein (K) is only twice as high for physical interactions (4.1) as for metabolic interactions (2.0) and the difference in clustering coefficients appears at least partly due to an intrinsic difference between physical and metabolic interaction networks.

Several metabolic pathways are completely retrieved, such as the arginine and the threonine biosynthesis pathways, which are connected only by predicted metabolic interactions (blue lines). The arginine biosynthesis pathway is depicted in Figure 4b. We find many known physical protein complexes as clusters densely connected by red lines, as has been previously shown in many integrative bioinformatics studies [18-21]. Interestingly, we now also observe the pathway interactions that exist between them. For example, in the upper right corner is the oxidative phosphorylation pathway. Members of the same complex have red lines (physical interactions) between them, whereas members of different complexes have blue lines (metabolic interactions) between them. Even though we derived the metabolic pathway interactions by identifying the regions in the landscapes that scored highly in a metabolic reference set, we still expect this class in addition to be general for other functional associations from other types of cellular pathways. Therefore, the blue lines between, for example, the exosome and the small nucleolar ribonucleoprotein complex, are not necessarily metabolic as in the case of the oxidative phosphorylation pathway, but rather other types of functional associations in which a substrate is passed on from one protein to another. Likewise, the oxidative stress cluster contains interactions between thioredoxin reductases and glutaredoxins. These proteins are, as far as is known, not part of the same pathway in the sense that they pass, for example, reducing equivalents to each other, but they are part of the same system.

## Discussion

It is perhaps logical in hindsight that we detect metabolic interactions in areas where both proteomic approaches report no co-purification while there are strong indications for co-regulation, but there are some important implications. We should use not only integrations based on the top scoring proteins but also non-scoring proteins. For the co-purification data this implies that the absence of a reported interaction is in fact the reflection of a cellular reality: in other words, we need physical protein interaction datasets where the negatives are really true negatives rather than the absence of results. Although the comparison of the Gavin *et al*. [3] and Krogan *et al*. [4] co-purification data reveals that both datasets still harbor some false negatives, a combined dataset of both comes close to having the perfect properties for our objective, and it is only since the publication of these data that a differential genomics approach as proposed here has become possible.

Another contribution in distinguishing metabolic from physical interactions comes from differential rates of evolution. We could not obtain the same level of differential ppv for the prediction of metabolic interactions in landscapes with the conserved co-expression set of Stuart and co-workers [11] as we did with a two-species orthologous conserved co-expression [10] because the first predicts mainly physical interactions. As the conserved co-expression set of Stuart *et al*. is based on four species and the other one on only two, we speculate that metabolic interactions are less conserved in evolution than physical interactions, which is consistent with results on the evolutionary modularity of metabolic pathways and protein complexes in biological systems [22]. The higher rate of evolution of metabolic interactions also explains that a very high level of conservation of gene neighborhood conservation or correlation of phylogenetic profiles indicates a physical interaction whereas intermediate levels are more indicative of metabolic interactions.

One striking observation is that we predict many more physical interactions than metabolic interactions. This difference might be easily explained by the fact that there are specific experimental methods to find physical interactions and no specific methods to find metabolic interactions. Even the shared genetic interactions, which we previously thought to be indicative of co-pathway membership, turn out to correlate mostly with physical protein interactions. Only co-expression data and the *in silico *prediction methods contain metabolic interactions mixed with physical interactions, making it hard to specifically extract metabolic interactions from omics data. Ultimately, it might even be the nature of metabolic interactions themselves that makes them less amendable to prediction: metabolic interactions are, by nature, indirect, and only in the case of linear pathways do the enzymes involved have the kind of mutual dependence that proteins in the same complex have, which might explain why the former leave a less strong signal in the genomics data than the latter. A weaker type of interaction between enzymes in the same pathway is also suggested by our observation that metabolic interactions are prevalent at intermediate degrees of gene order conservation or correlation between phylogenetic profiles while high levels of gene order conservation correlate with physical interactions.

It is of course tempting to combine more than two types of omics data. There are, however, two reasons why we here explore pairs of evidence types rather than the multidimensional evidence landscape given by all evidence types simultaneously. Firstly, visual inspection of differential ppv plots is still possible in two dimensions but becomes more troublesome in higher dimensions. Secondly, and more importantly, overlapping all evidence types at the same time results in very small numbers of protein pairs in each multidimensional volume in the reference sets, which in turn hampers the reliable calculation of prediction ppv.

As an extension to this work we would like to specifically predict more than only two types of interactions. One type of interaction that we can not predict is a kinase-target interaction; the prediction of these kinds of interactions is a field on its own and requires integration of many more types of prediction methods and data, such as sequence data [23]. Furthermore, for the type of method we use here it is necessary to have reference sets that are of high quality and at the same time cover many protein pairs. For transient physical interactions, such reference sets are not available at the moment, although they might become available in the near future.

Protein relations predicted by our computational integration should be less laborious to experimentally test, because they prioritize the usability of various assays for biochemical verification. For example, it would be disingenuous to verify our metabolic relations by CoIP. In general, we expect that novel ways of integration and the advent of more and more types of omics data will allow the further development of approaches to increase the specificity and to extract more qualitative data on the nature of protein interactions.

## Conclusion

When predicting interactions between genes it is essential to specify the type of interaction that is predicted to allow biological interpretation. Some data types are already specific for the type of interaction, for example, ChIP-on-chip data of transcription factors is indicative of regulatory interactions and co-purifications are specific for physical interactions. However, co-regulation, correlated expression, shared genetic interactions and *in silico *interactions are not intrinsically specific to any type of interaction. Here we have shown that although some datasets do contain a high level of metabolic interactions at intermediate scores, it is not possible to reliably predict metabolic interactions from them. However, by combining the datasets in ways that examine the whole evidence landscape and not only the highest scoring protein pairs in both datasets we can find specific predictions; for example, by taking protein pairs whose co-expression is evolutionarily conserved but that never co-purify in two comprehensive protein-protein interaction datasets, we can label these predicted interaction as metabolic interactions. This is a first step towards improved biological interpretation of gene networks generated from the integration of high throughput data.

## Materials and methods

### Evidence types

#### Protein-protein interactions

We downloaded the yeast protein complex purifications published by Gavin and co-workers [3] and recalculated the SA scores that reflect the likelihood of interaction to include also proteins that were purified only once. Protein pairs that weree never co-purified but were both purified at least once received a SA score of zero. We also downloaded the protein complex purifications of Krogan and co-workers [4]. These authors produced a different interaction score per protein pair, which was optimized to overlap with protein complexes from the MIPS database. To have a reference set-independent score we calculated SA scores based on the purifications of Krogan *et al*. Protein pairs that were never found together in a purification but were purified at least once were given a score of zero. As a third set we took the sum of SA scores of all protein pairs occurring in both protein-protein interaction datasets. Scored yeast-two-hybrid interactions were obtained from the STRING database [24].

#### *In silico *predictions

*In silico *predictions of functional interactions were obtained from the STRING database [24]. From this database we took the co-occurrence scores based on phylogenetic profiles of COGs and gene neighborhood conservation also based on COGs. The scores were transferred from pairs of COGs to pairs of *S. cerevisiae *genes. If more than three yeast genes belonged to the same COG, the score was considered ambiguous and was removed from the dataset.

#### Conserved co-expression

We used two multi-species conserved co-expression datasets; co-expression conservation between human, yeast, fly and worm [11] and between yeast and worm [10]. We also used co-expression conservation between pairs of paralogs [10] in yeast. For the two-species conservation we took the maximum expression correlation of all pairs of orthologs and averaged this maximum with the expression correlation of the gene pair itself. For paralogous conservation we took the maximum expression correlation between all parallel duplicated gene pairs and averaged this maximum with the expression correlation of the gene pair itself.

#### Co-regulation

Co-regulation was assessed by combining correlated mRNA expression profiles with similarity in bound regulators to the gene promoter. Rick Young's lab made a comprehensive survey of the gene regulatory network in yeast [25]. We took a cut-off of 0.01 for binding of a transcription factor to a promoter based on the raw ChIP-on-chip data and divided the number of shared transcription factors between two genes *Ni, j *by the geometric average of the total number of transcription factors bound by each of the two genes *T *resulting in a co-regulation score *Sij*:

$$S_{ij}=\frac{N_{ij}}{\sqrt{T_{i}^{2}+T_{j}^{2}}}$$

Gene pairs that share a promoter were excluded. To increase the reliability of the co-regulation signal, we multiplied the correlation in binding profile by the correlation in mRNA expression profile based on a large-scale expression dataset in yeast [11], that is:

*Snew ij *= *rij *× *Sij*

where *rij *is the expression correlation of gene *i *and *j*.

#### Synthetic lethality

A set of synthetic lethal and synthetic sick interactions were downloaded from the *Saccharomyces *Genome Database [26]. It was found earlier that genetic interactions [6] on their own are only marginally useful for predicting direct interactions, but shared genetic interactions do indicate involvement in similar pathways [27]. We corrected the number of shared genetic interactions *Ni, j *by the geometric average of total interactions *T *per protein, exactly the same as for the co-regulation score.

### Reference sets

We downloaded known complexes from MIPS [28] and removed all categories containing the terms 'other' or 'predicted'. Removal of the predicted category was especially acute, because these contain complexes derived from purified complexes identified by mass-spectrometry from earlier high-throughout publications from the same groups that produced the Krogan *et al*. and the Gavin *et al*. datasets. We took complexes at the lowest level of definition. Protein pairs that are in the same complex are positive examples, and protein pairs that are in different complexes are negative examples. The positive and negative examples constitute the physical interaction reference set.

From the KEGG database [29] we took all metabolic maps with indices smaller than 2,000. Maps with higher index are not metabolic and contain other processes, including many that consist of a single protein complex. Positive examples are all protein pairs that co-occur on a metabolic map, and negative examples are all protein pairs that do not co-occur on a metabolic map but are, nevertheless, present in the metabolic maps of KEGG. In order to not have any physical interactions in our metabolic reference set, we removed all protein pairs with the same EC number and removed all protein pairs that are part of the same complex according to SGD/GO annotation [30,31] or MIPS. Together, the positive and negative examples form the metabolic interaction reference set.

Cytoplasmic ribosomal proteins were removed from all reference sets and datasets. As they confer very many pair-wise interactions, including them would bias all statistics towards ribosomes.

### ppv and differential ppv

The conserved co-expression values of the Kim lab [11] were rescaled by transforming the -log(*P*-value) to scores between 0 and 1, such that high scores correspond to more likely interactions. All other scores were rescaled to scores between 0 and 1 by a linear transformation. In the score-ppv plots for each set we calculated ppv based on intervals with bin width 0.025. In the evidence landscape plots, we plotted two datasets against each other in a heat map-like fashion and color squares according to their differential ppv (see below). Squares were made with sides of 0.05; if a square contained fewer than two true positives, a larger square with sides 0.1 was made to avoid high performance scores based on very few examples.

Physical interaction ppv (ppv *phys*) was calculated as the number of true positives of the physical interaction reference set divided by the number of true positives plus false positives of both reference set sets in that bin (Table 2). Metabolic interaction ppv (ppv *meta*) was calculated as the number of true positives of the metabolic interaction reference set divided by the number of true positives plus false positives of both reference sets in that bin. In order to score for how well a given region/square bin in the evidence landscape predicts either type of interaction, we computed what we here call the differential ppv (ppv *diff*).

**Table 2:** True positives and false positives

	Positive metabolic	Negative metabolic	Positive physical	Negative physical
Present in bin	TP *meta*	FP *meta*	TP *phys*	FP *phys*

ppv *meta *= TP *meta*/(TP *meta *+ FP *meta *+ TP *phys *+ FP *phys*)

ppv *phys *= TP phys/(TP *meta *+ FP *meta *+ TP *phys *+ FP *phys*)

ppv *diff *= ppv *meta *- ppv *phys*

Differential ppv is computed by subtracting the ppv *phys *from ppv *meta*. This means that if a region scores equally well in both reference sets (be it very poor or very well), it has a zero differential ppv, reflecting the inability of this region to differentiate between metabolic and physical interactions. However, if it is very accurate in predicting metabolic relations but unable to accurately predict physical interactions, it has a very high differential ppv and, vice versa, a very negative value reflects specificity for physical interactions.

### Logistic regression

We took all gene pairs that fell into the reference sets and took as a binary dependent variable the absence or presence of a known interaction. Again, for metabolic interactions the gene pairs of the physical interaction reference set were added as gene pairs with an absent interaction and for physical interactions the gene pairs of the metabolic interaction reference set were added as gene pairs with an absent interaction. The scores of the 'omics' datasets were, in turn, considered as the continuous independent variable to fit a logit function. The intercepts (*a*) and coefficients (*b*) are reported in Table 1. An approximation of the R^2 ^value was calculated as:

R^2 ^= (null variance - residual variance)/(null variance)

### Adding specificity to predicted interactions

We took all gene pairs that fell into squares with differential ppv larger than 0.95 and at least five true positive metabolic interactions and called them 'predicted metabolic interactions'. We selected all gene pairs that fell into squares with differential ppv smaller than -0.95 and at least five true positive physical interactions and called them 'predicted physical interactions'.

### Software

Figure 2 was made using xmgrace [32]. The panels of Figure 3 were made using R [33]. The network of predicted interactions was visualized using cytoscape [34].

## Abbreviations

ChIP-chip, chromatin imuno purification followed by chip; CoExp, correlated expression; CoExp2Sp, correlated expression in two species; CoExp4Sp, correlated expression in four species; CoOccur, phylogenetic cooccurrence; GenNeigh, gene neighborhood conservation; FP, false positive; ppv, positive predictive value; SA, socio-affinity; TP, true positive.

## Authors' contributions

VvN and BS contributed to the data acquisition, design of the analysis, analysis and interpretation of the data and drafting and revising the manuscript. MH contributed to the design of the analyses and revising the manuscript. All authors have read and approved the final manuscript.

## Additional data files

The following additional data are available with the online version of this paper. Additional data file 1 is a correlation matrix that provides the correlation between the scores of all pairs of omics datasets.

## Supplementary Material

Additional date file 1 This correlation matrix provides the correlation between the gene pair scores of all pairs of omics datasets. For each pair of datasets, the correlations are based on gene pairs that are present in both datasets. High values in this matrix show that the scores in omics datasets can not be used as independent variables in a multivariate logistic regression. Click here for file
